# Epac induces ryanodine receptor-dependent intracellular and inter-organellar calcium mobilization in mpkCCD cells

**DOI:** 10.3389/fphys.2023.1250273

**Published:** 2023-08-29

**Authors:** Kay-Pong Yip, Luisa Ribeiro-Silva, Byeong Cha, Timo Rieg, James S. K. Sham

**Affiliations:** ^1^ Department of Molecular Pharmacology and Physiology, Morsani College of Medicine, University of South Florida, Tampa, FL, United States; ^2^ Hypertension and Kidney Research Center, Morsani College of Medicine, University of South Florida, Tampa, FL, United States; ^3^ James A. Haley Veterans’ Hospital, Tampa, FL, United States; ^4^ Division of Pulmonary and Critical Care Medicine, Johns Hopkins University School of Medicine, Baltimore, MD, United States

**Keywords:** intracellular calcium stores, Epac, mitochondria-associated membrane, aquaporin-2, ryanodine receptor

## Abstract

Arginine vasopressin (AVP) induces an increase in intracellular Ca^2+^ concentration ([Ca^2+^]_i_) with an oscillatory pattern in isolated perfused kidney inner medullary collecting duct (IMCD). The AVP-induced Ca^2+^ mobilization in inner medullary collecting ducts is essential for apical exocytosis and is mediated by the exchange protein directly activated by cyclic adenosine monophosphate (Epac). Murine principal kidney cortical collecting duct cells (mpkCCD) is the cell model used for transcriptomic and phosphoproteomic studies of AVP signaling in kidney collecting duct. The present study examined the characteristics of Ca^2+^ mobilization in mpkCCD cells, and utilized mpkCCD as a model to investigate the Epac-induced intracellular and intra-organellar Ca^2+^ mobilization. Ca^2+^ mobilization in cytosol, endoplasmic reticulum lumen, and mitochondrial matrix were monitored with a Ca^2+^ sensitive fluorescent probe and site-specific Ca^2+^ sensitive biosensors. Fluorescence images of mpkCCD cells and isolated perfused inner medullary duct were collected with confocal microscopy. Cell permeant ligands of ryanodine receptors (RyRs) and inositol 1,4,5 trisphosphate receptors (IP_3_Rs) both triggered increase of [Ca^2+^]_i_ and Ca^2+^ oscillations in mpkCCD cells as reported previously in IMCD. The cell permeant Epac-specific cAMP analog Me-cAMP/AM also caused a robust Ca^2+^ mobilization and oscillations in mpkCCD cells. Using biosensors to monitor endoplasmic reticulum (ER) luminal Ca^2+^ and mitochondrial matrix Ca^2+^, Me-cAMP/AM not only triggered Ca^2+^ release from ER into cytoplasm, but also shuttled Ca^2+^ from ER into mitochondria. The Epac-agonist induced synchronized Ca^2+^ spikes in cytosol and mitochondrial matrix, with concomitant declines in ER luminal Ca^2+^. Me-cAMP/AM also effectively triggered store-operated Ca^2+^ entry (SOCE), suggesting that Epac-agonist is capable of depleting ER Ca^2+^ stores. These Epac-induced intracellular and inter-organelle Ca^2+^ signals were mimicked by the RyR agonist 4-CMC, but they were distinctly different from IP_3_R activation. The present study hence demonstrated that mpkCCD cells retain all reported features of Ca^2+^ mobilization observed in isolated perfused IMCD. It further revealed information on the dynamics of Epac-induced RyR-dependent Ca^2+^ signaling and ER-mitochondrial Ca^2+^ transfer. ER-mitochondrial Ca^2+^ coupling may play a key role in the regulation of ATP and reactive oxygen species (ROS) production in the mitochondria along the nephron. Our data suggest that mpkCCD cells can serve as a renal cell model to address novel questions of how mitochondrial Ca^2+^ regulates cytosolic Ca^2+^ signals, inter-organellar Ca^2+^ signaling, and renal tubular functions.

## Introduction

Physiological concentration of arginine vasopressin (AVP) induces intracellular Ca^2+^ mobilization in form of oscillation in isolated perfused rat inner medullary collecting duct (IMCD) (Yip, 2002). Confocal fluorescence microscopy revealed that each IMCD cells have their own unique oscillatory frequency and amplitude. Such Ca^2+^ mobilization is essential for the associated apical exocytosis, as intracellular Ca^+2^ chelators inhibit both AVP-induced Ca^2+^ mobilization and apical exocytosis in perfused IMCD. AVP exerts its actions via binding of the V_2_-receptors to stimulate adenylate cyclase and cAMP production in IMCD cells (Knepper and Inoue, 1997), the latter is mediated by adenylyl cyclase 6 (Rieg et al., 2010). It has traditionally been thought that cAMP activates the protein kinase A (PKA)-dependent signaling pathway to mediate AVP-regulated osmotic water permeability of IMCD. However, our previous study found that PKA inhibitors did not prevent AVP-induced Ca^2+^ mobilization and oscillation. Instead, the cAMP analog 8-pCPT-2′-O-Me-cAMP, which specifically activates exchange protein directly activated by cAMP (Epac) but not PKA, triggered intracellular Ca^2+^ mobilization and apical exocytosis of aquaporin-2 (AQP2) in perfused IMCD (Yip, 2006). Moreover, flash photolysis of caged cADP-ribose (an endogenous ligand of ryanodine receptors) activated Ca^2+^ oscillations resembling AVP-induced Ca^2+^ response (Yip and Sham, 2011). Previous studies showed that Ca^2+^ release from ryanodine receptors (RyRs) is essential in AVP-mediated AQP2 trafficking (Chou et al., 2000; Yip, 2002), and the process is independent of the phosphoinositol signaling pathway (Chou et al., 1998). AVP could also trigger Ca^2+^ influx via the store-operated Ca^2+^ entry (SOCE) mechanism. It was concluded that AVP-induced Ca^2+^ oscillation in IMCD is mediated by an Epac-dependent mechanism through the interplay of Ca^2+^ release from ryanodine receptors and a Ca^2+^ influx mechanism involving SOCE (Yip and Sham, 2011). Epac-induced Ca^2+^ release from RYRs-gated Ca^2+^ stores have been reported in other cell types. In cardiac myocytes, Epac-activation enhances RYR activity through protein kinase C_epsilon_ and Ca^2+^/calmodulain kinase II (CaMKII)-dependent phosphorylation of RYRs (Pereira et al., 2007; Oestreich et al., 2009), leading to SR Ca^2+^ leak and arrhythmia (Pereira et al., 2013; Li et al., 2017; Pereira et al., 2017). Epac-induced activation of RYRs also causes membrane hyperpolarization and relaxation of mesenteric arteries through Ca^2+^-sensitive K^+^ channel activation (Roberts et al., 2013).

Murine principal kidney cortical collecting duct (mpkCCD) cells are commonly used cell model used for transcriptomic and phosphoproteomic studies of AVP-signaling in kidney collecting ducts (Rinschen et al., 2010; Huling et al., 2012; Sandoval et al., 2013; Yang et al., 2022; Park et al., 2023). It is assumed that mpkCCD cell retains the feature of intact collecting duct cell in AVP-induced signaling events of AQP2 trafficking. We have demonstrated cAMP-dependent vectorial trafficking and exocytosis of AQP2 tagged with photoactivable fluorescent protein in mpkCCD cells at real time (Yip et al., 2015). It has also been shown in mpkCCD cells that Wnt5A, an endogenous ligand of the non-canonical branch of the Wnt pathway, is capable of inducing AQP2 apical expression and trafficking via basolateral Fzd receptors-mediated Ca^2+^ mobilization without activation of cAMP/PKA signal pathway (Ando et al., 2016). These observations highlight the potential of targeting Ca^2+^ pathways to ameliorate polyuria associated with nephrogenic diabetes insipidus (Mortensen et al., 2020). However, there is no information on the mechanisms underlying the dynamics of intracellular Ca^2+^ mobilization in mpkCCD cells. It is also unclear whether mpkCCD cells retain the specific properties of Ca^2+^ mobilization observed in perfused IMCD. In the present study, we sought to verify mpkCCD cells as a reliable model representing collecting duct cells for the study of the intracellular Ca^2+^ stores, the mechanisms of Ca^2+^ release, and extracellular Ca^2+^ influx. Moreover, special emphasis has been placed on the Epac-induced temporal relationship of Ca^2+^ dynamics in the cytosol, endoplasmic reticulum (ER) and mitochondria. Our results demonstrate that mpkCCD cells display similar characteristics of intracellular Ca^2+^ mobilization observed in intact cells of collecting duct, and that the Epac agonist triggered intracellular Ca^2+^ mobilization and oscillation are mediated by RyR-gated Ca^2+^ release and SOCE associated with reciprocal decrease of Ca^2+^ content in the ER. Moreover, the Epac agonist can effectively shuttle ER luminal Ca^2+^ to both the cytosol and mitochondrial matrix.

## Materials and methods

### Cell culture

Experiments were performed on a male mouse CCD principal cell line (mpkCCD_C14_, kindly provided by Dr. Douglas Eaton, Emory University) grown in AVP-free culture medium. Cells were maintained in a 1:1 mixture of DMEM/Ham’s F12 medium with phenol red (Gibco), supplemented with dexamethasone (50 nM), triiodothyronine (1 nM), selenium (60 nM), insulin (5 μg/mL), mouse EGF (10 ng/mL), transferrin (5 μg/mL), and 2% fetal calf serum in a humidified atmosphere with 5% CO_2_ at 37°C. mpkCCD cells between 20 and 30 passages were grown on collagen coated glass bottom dish prior to the experiments.

### Monitoring of cytosolic Ca^2+^

mpkCCD cells grown on collagen coated glass bottom dish (MatTek) were loaded with cell permeant Ca^2+^ sensitive fluorescence probe (Cal-520/AM, 5 μM, AAT Bioquest) in phenol red free medium (1:1 mixture of DMEM/Ham’s F12 medium, Gibco) for 30 min at 37°C, followed by 20 min for de-esterification. Fluorescent images were collected with a Leica TCS SP5 confocal imaging system using water immersion objective lens (×63, N.A. 1.2) equipped with environmental chamber. Cal-520 was excited at 488 nm, and the emission was collected with a spectral window of 495–530 nm at 1 Hz. The spatial and temporal variations of [Ca^2+^]_i_ in individual cells were measured from the stored images with Leica Application Suite Advanced Fluorescence software as reported previously (Yip and Sham, 2011). Store-operated calcium entry (SOCE) was induced by thapsigargin (10 µM) in calcium-free Hanks’ Balanced Salt Solution (Gibco) following by re-addition of 2 mM Ca^2+^ in the extracellular buffering solution.

### Monitoring of calcium in ER and mitochondria with biosensors

To monitor ER [Ca^2+^] ([Ca^2+^]_ER_) or mitochondrial [Ca^2+^] ([Ca^2+^]_MITO_) simultaneously with cytosolic [Ca^2+^]_i_, mpkCCD cells were transfected with either ER Ca^2+^ biosensor R-CEPIA1er (Addgene Plasmid #58216, λ_ex_: 543 nm, λ_em_: 560–600 nm) or mitochondrial Ca^2+^ biosensor mito-RCaMP1h (Addgene Plasmid #105013, λ_ex_: 543 nm, λ_em_: 560–600 nm) (Suzuki et al., 2014). Cells were seeded at 6 × 10^4^ cells/cm^2^ on collagen coated glass bottom dishes for 24 h before transfection. Cells were transfected with Lipofectamine (0.5 µg DNA/1 × 10^5^ cells) for 24 h according to manufacturer’s instruction. Studies were performed in transfected cells from 48 to 72 h after transfection. Cytosolic [Ca^2+^]_i_ was monitored simultaneously with cell permeant Ca^2+^ sensitive fluorescence probe (Cal-520/AM) in the transfected cells incubating with phenol red free medium (1:1 mixture of DMEM/Ham’s F12 medium, Gibco). To monitor ER-mitochondrial Ca^2+^ transfer in mpkCCD cells, cells were co-transfected with the ER Ca^2+^ biosensor (G-CEPIA1er, Addgene Plasmid #58215, λ_ex_: 488 nm, λ_em_: 510–540 nm) and mitochondrial biosensor mito-RCaMP1h. Fluorescent images were collected with the respective laser lines for excitation and spectral windows for emission using the Lecia TCS SP5 imaging system.

### Perfusion of rat inner medullary collection duct (IMCD)

All animal experimentation was conducted in accordance with the National Institutes of Health Guide for Care and Use of Laboratory Animals (National Institute of Health, Bethesda, MD) and was approved by the University of South Florida Institutional Animal Care and Use Committee (PROTOCOL #R3982). IMCDs were isolated from male Sprague-Dawley rats and perfused as described previously (Yip, 2002). Cytosolic [Ca^2+^]i in perfused IMCD was monitored with fluo-4/AM (5 μM, Invitrogen) in individual IMCD cells. Confocal fluorescent images of IMCD were collected and analyzed as reported previously (Yip, 2002).

### Chemicals

6-Bnz-cAMP-AM, Me-cAMP-AM (8-pCPT-2′-O-Me-cAMP-AM), and ESI-09 were purchased from Biolog (Germany). Ryanodine, SKF-96365, and Xestospongin C were purchased from MilliporeSigma (Burlington, MA). Bt_3_-Ins(1,3,5)P_3_/AM was purchased from SiChem. ATP, 4-CMC (4-Chloro-m-cresol), H-89, and thapsigargin were from Sigma-Aldrich (St. Louis, MO).

### Data analysis

Time series of fluorescence emission variations in individual mpkCCD cells were extracted and normalized with respective to the base line from stored XYT images. Time series of Cal-520 emission from individual cells were sampled at 1 Hz for spectral analysis. Each time series was subjected to linear trend removal. 512 or 1,024 data points were used to calculate the power spectrum with an algorithm based on Fast Fourier Transform (Yip et al., 1991). Results were reported as mean ± standard error. Statistical significance was calculated by using student’s *t* tests for paired or unpaired data and considered significant when *p* < 0.05.

## Results

### Intracellular Ca^2+^ mobilization in mpkCCD cells

**FIGURE 1:**
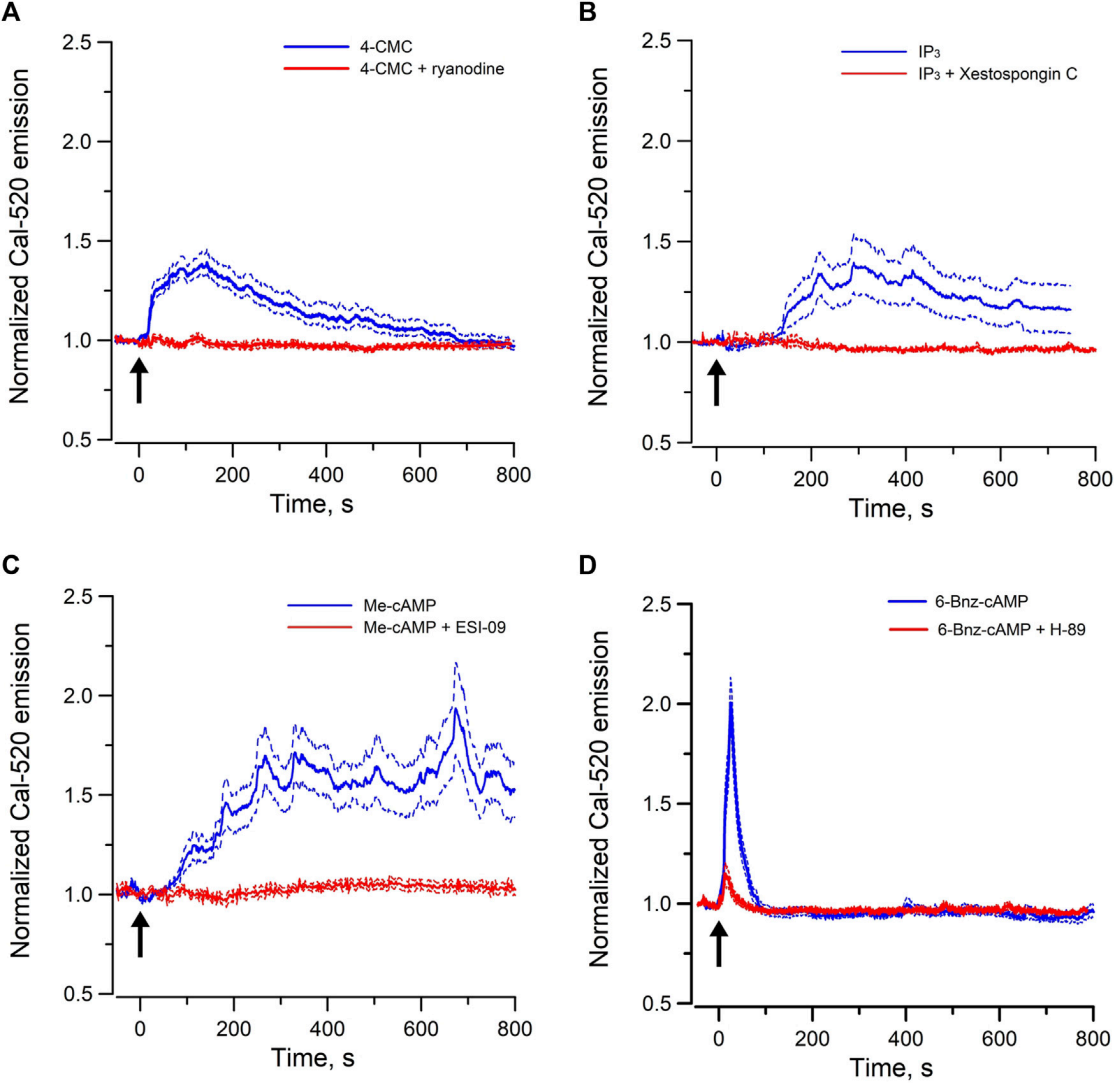
Mean normal time courses of Ca^2+^ mobilization in mpkCCD cells induced by **(A)** cell permeant ryanodine receptor agonist (50 µM 4-CMC, 54 cells/4 dishes), and **(B)** cell permeant IP_3_ receptor agonist (200 µM Bt_3_-Ins(1,3,5)P_3_/AM, 49 cells/4 dishes), **(C)** cell permeant Epac-agonist (40 µM Me-cAMP/AM, 92 cells/6 dishes), and **(D)** cell permeant PKA-agonist (40 µM 6-Bnz-cAMP/AM, 95 cells/6 dishes). Ryanodine (50 μM, 26 cells/3 dishes), Xestospongin C (10 μM, 35 cells/3 dishes), ESI-09 (25 μM, 36 cells/3 dishes), and H-89 (10 μM, 31 cells/3 dishes) were used as the corresponding receptor blockers or antagonists. Arrow (↑) indicates application of agonist in each time course. Dash lines are standard error.

RyRs are mainly expressed in the sarcoplasmic reticulum of skeletal, cardiac and smooth muscle cells, whereas inositol 1,4,5 trisphosphate receptors (IP_3_Rs) are the predominant Ca^2+^ release channels of the ER in non-excitable cells. We have previously shown that endogenous agonist of RyRs and IP_3_Rs triggered Ca^2+^ oscillations in individual cells of perfused IMCD, indicating both functional RyR-gated and IP_3_R-gated intracellular Ca^2+^ stores are present in IMCD cells (Yip and Sham, 2011). To test whether these Ca^2+^ stores are intact in mpkCCD cells, changes in [Ca^2+^]_i_ were monitored with Ca^2+^ sensitive fluorescence probe when cells were stimulated with 4-CMC (a cell permeant RyR agonist) or Bt_3_-Ins(1,3,5)P_3_/AM (a cell permeant agonist of IP_3_R). Ryanodine was used as blocker of RyRs, and Xestospongin C was used for IP_3_Rs. Both agonists triggered robust intracellular Ca^2+^ mobilization and oscillations in mpkCCD cells (Figures 1A, B). The 4-CMC-induced Ca^2+^ response was almost instantaneous, compared to the delayed Bt_3_-Ins(1,3,5)P_3_/AM triggered Ca^2+^ response (∼100–150 s). The 4-CMC-induced Ca^2+^ transient was completed at 800 s, whereas the IP_3_-triggered response was more sustained. The delayed IP_3_–induced Ca^2+^ response in the Ca^2+^ responses were possibly related to the rate of membrane permeation and de-esterification of Bt_3_-Ins(1,3,5)P_3_/AM. The differences in the kinetic profiles triggered by the two stores could be due to differences in the potency of the agonists, inactivation kinetics of the receptors, and the depletion or replenishment of the SR Ca^2+^ stores. Both 4-CMC and Bt_3_-Ins(1,3,5)P_3_/AM-induced Ca^2+^ responses were completely abolished in the presence of their respective receptor blockers. These observations confirmed that mpkCCD cells possess both functional RyR- and IP_3_R-gated Ca^2+^ stores as in IMCD.

**FIGURE 2:**
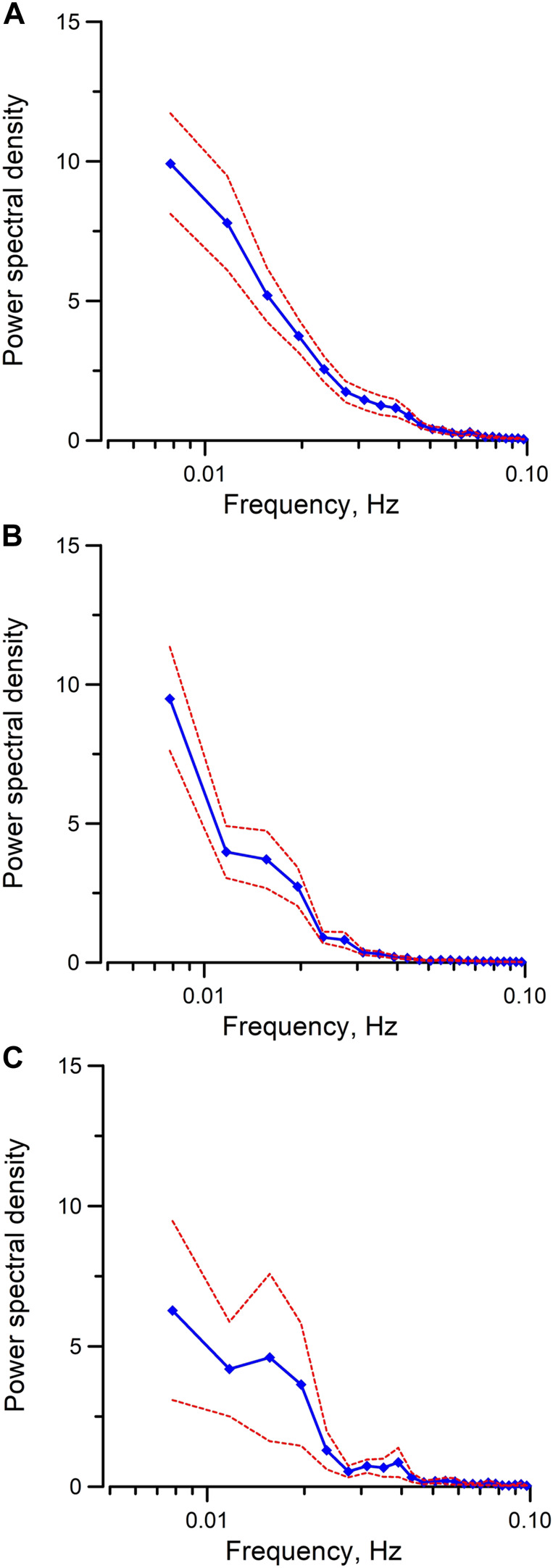
Mean power spectral spectra of cytosolic Ca^2+^ oscillations induced by **(A)** Me-cAMP/AM, **(B)** 4-CMC, **(C)** Bt_3_-Ins(1,3,5)P_3_/AM in mpkCCD cells. The same time series presented in Figure 1 were used for spectral analysis. By integrating the spectral power density from 0.03 Hz to 0.1 Hz in each individual power spectrum, the mean integrated spectral power density is significantly higher (*p* < 0.05) when cytosolic calcium oscillations were induced by Me-cAMP/AM than those induced by 4-CMC, or by Bt_3_-Ins(1,3,5)P_3_/AM. Dash lines are standard error.

To examine the Epac-dependent Ca^2+^ mobilization and oscillation (Yip, 2006), mpkCCD cells were stimulated with Me-cAMP/AM. Me-cAMP/AM is a cell permeant cAMP analog which activates specifically Epac but not PKA. Me-cAMP/AM triggered larger and sustained intracellular Ca^2+^ mobilization and oscillations in mpkCCD cells compared to those induced by 4-CMC and IP_3_ (Figure 1C). The Ca^2+^ response was blocked by ESI-09, an inhibitor of Epac1 and Epac2. These two Epac isoforms are expressed in IMCD and mpkCCD cells (Li et al., 2008; Kortenoeven et al., 2012). In contrast, 6-Bnz-cAMP/AM, a cell permeant cAMP analog which activates PKA but not Epac, triggered only a brief transient Ca^2+^ mobilization without Ca^2+^ oscillation (Figure 1D). The brief Ca^2+^ transient was effectively attenuated by the PKA inhibitor H-89. These observations suggested that activation of the Epac-dependent signal pathway elicits sustained Ca^2+^ mobilization and oscillation in mpkCCD cells. Spectral analysis was further applied to individual time series of Ca^2+^ signals of individual mpkCCD cells to characterize the oscillatory frequencies and the power of the Ca^2+^ oscillations. The mean power spectral density induced by Me-cAMP/AM, 4-CMC, and Bt_3_-Ins(1,3,5)P_3_/AM, were shown in Figure 2. All three mean power spectra had broad distribution over a range of frequencies. Most of the oscillations were confined in frequency range of 0.007–0.1 Hz, which are consistent with observations from intact IMCD cells of perfused IMCD (Yip, 2002; Yip and Sham, 2011). The power of Me-cAMP/AM, 4-CMC, and Ins(1,3,5)P_3_/AM-induced Ca^2+^ oscillations were similar, except Me-cAMP/AM induced Ca^2+^ oscillation had more power at the higher frequencies (0.03–0.1 Hz frequency range).

### Epac and RyR-agonist mobilize ER Ca^2+^ for cytosolic Ca^2+^ oscillation and ER-mitochondrial Ca^2+^ transfer in mpkCCD cells

**FIGURE 3:**
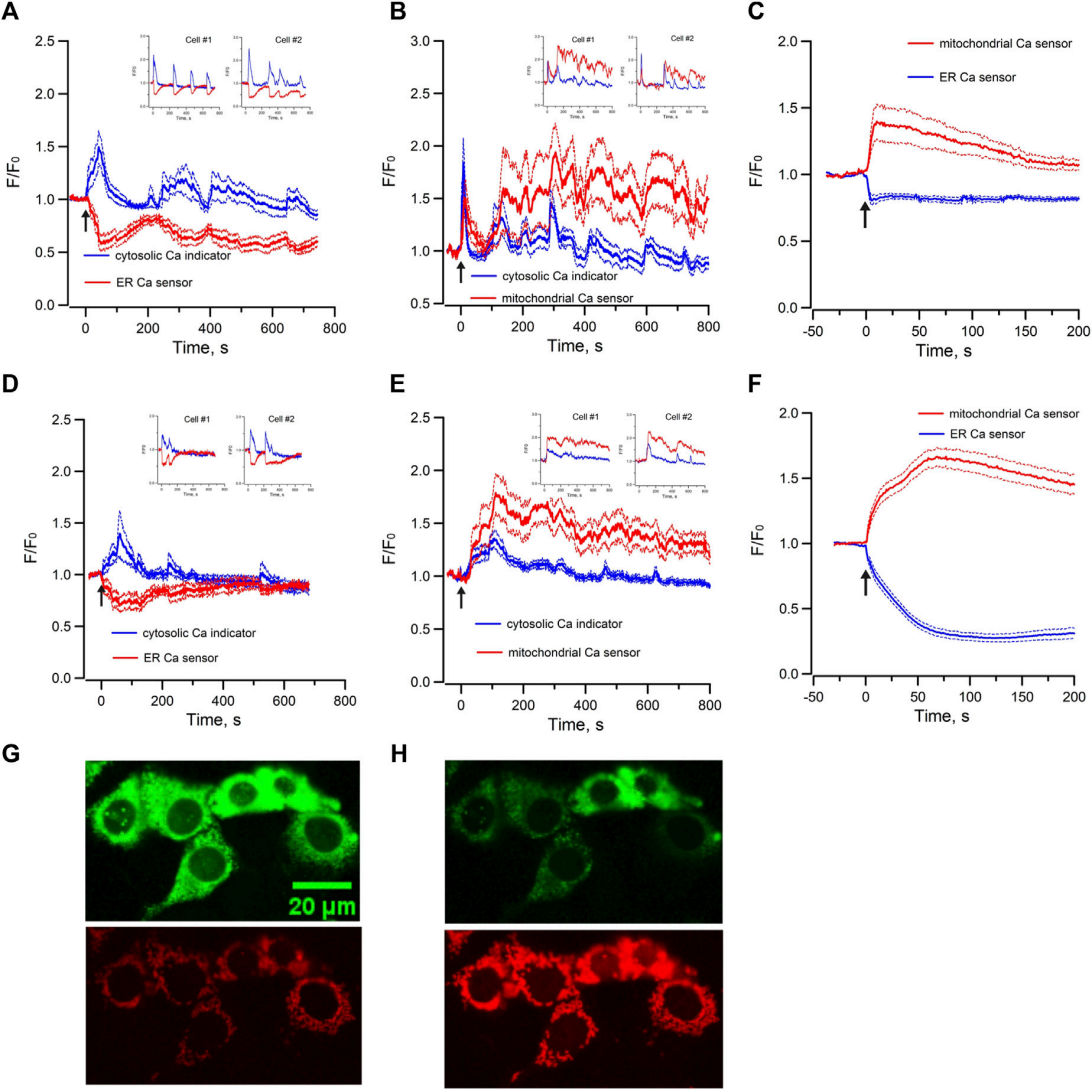
Mean normalized time courses of simultaneous changes in **(A)** cytosolic Ca^2+^ and ER luminal Ca^2+^ (15 cells/3 dishes), **(B)** cytosolic Ca^2+^ and mitochondrial matrix Ca^2+^ (20 cells/3 dishes) and **(C)** ER luminal Ca^2+^ and mitochondrial matrix Ca^2+^ (36 cells/7 dishes) induced by 40 µM Me-cAMP/AM in mpkCCD cells. Corresponding changes induced by 50 µM 4-CMC are shown in **(D)** cytosolic Ca^2+^ and ER luminal Ca^2+^ (13 cells/3 dishes), **(E)** cytosolic Ca^2+^ and mitochondrial matrix Ca^2+^ (21 cells/3 dishes), and **(F)** ER luminal Ca^2+^ and mitochondrial matrix Ca^2+^ 55 cells/9 dishes). mpkCCD cells expressing both ER biosensor (green) and mitochondrial biosensor (red) before **(G)** and after **(H)** exposure to RyR agonist 4-CMC. The Arrow (↑) indicates application of agonist in each time course. Inserts are tracings from individual cells with multiple Ca^2+^ spikes. F/F_0_ is the fractional change in fluorescence emission of the fluorescent probe or biosensor. Dash lines are standard error.

To further characterize Epac-dependent activation of ER Ca^2+^ stores in mpkCCD cells, the temporal variations of cytosolic Ca^2+^ and ER luminal Ca^2+^ were monitored simultaneously with the Ca^2+^ sensitive-fluorescence probe Cal-520/AM and the ER luminal Ca^2+^ biosensor R-CEPIA1er, respectively. Me-cAMP/AM triggered an increase of cytosolic Ca^2+^ which was associated with a synchronized decrease in ER luminal Ca^2+^ content (Figure 3A). The oscillations in cytosolic Ca^2+^ were mirror images of those in ER luminal Ca^2+^. These observations suggested that the Epac-induced increase of cytosolic Ca^2+^ was due to release of Ca^2+^ from ER intracellular Ca^2+^ stores, and the cyclic variations in luminal ER Ca^2+^ content were likely due to ER Ca^2+^ depletion and refilling by Ca^2+^ uptake via the sacroplasmic/endoplasmic reticular Ca^2+^-ATPase (SERCA). RyR-agonist 4-CMC triggered similar response in mpkCCD cells (Figure 3D), indicating that Ca^2+^ release from RyR-gated stores generates cytosolic and ER Ca^2+^ signals comparable to those of Epac activation, congruent with reports that Epac triggered Ca^2+^ release via RyRs in IMCD and cardiomyocytes (Yip, 2006; Valli et al., 2018).

Mitochondrial Ca^2+^ concentration is important for the regulation of mitochondrial functions, and it is regulated by local Ca^2+^ concentration in the proximity of Ca^2+^ release channels of ER (Rizzuto et al., 2012; Csordas et al., 2018). To test whether there is mitochondrial matrix Ca^2+^ uptake during Epac-mediated Ca^2+^ mobilization in mpkCCD cells, the variations of cytosolic Ca^2+^ and mitochondrial matrix Ca^2+^ were monitored simultaneously with Ca^2+^ sensitive fluorescence probe and the mitochondrial matrix Ca^2+^ biosensor mito-RCaMP1h. Application of Me-cAMP/AM to mpkCCD cells activated multiple synchronized Ca^2+^ spikes in the cytosol and mitochondrial matrix (Figure 3B). These observations suggested that the Epac-agonist not only triggers release of ER Ca^2+^ to the cytosol, but also shuttles ER Ca^2+^ into the mitochondria. RyR-agonist 4-CMC triggered a similar response with synchronized Ca^2+^ spikes in the cytosol and mitochondrial matrix (Figure 3E). To determine the temporal relationship between ER luminal Ca^2+^ and mitochondrial matrix Ca^2+^, the ER luminal Ca^2+^ biosensor G-CEPIA1er and the mitochondrial matrix Ca^2+^ biosensor mito-RCaMP1h were co-expressed in mpkCCD cells. Me-cAMP/AM triggered a decrease in ER luminal Ca^2+^, which was associated with a concomitant increase in mitochondrial matrix Ca^2+^ (Figure 3C). RyR-agonist 4-CMC triggered a similar response (Figure 3F), indicative of effective RyR-coupled ER-mitochondrial Ca^2+^ transfer in mpkCCD cells. Figures 3G, H are fluorescence images of ER (green) and mitochondria (red) demonstrating Ca^2+^ transfer from ER to mitochondria induced by RyR agonist 4-CMC.

**FIGURE 4:**
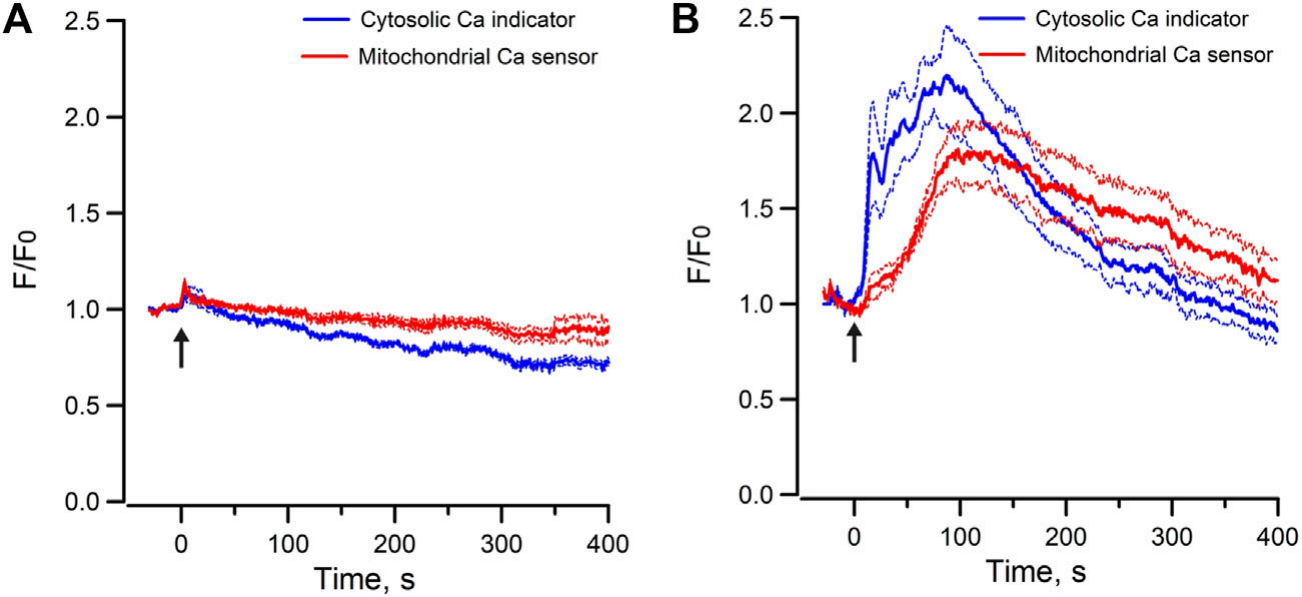
Mean normalized time courses of simultaneous changes in cytosolic Ca^2+^ and mitochondrial matrix Ca^2+^ induced by 40 µM Me-cAMP/AM in mpkCCD cells preincubated with **(A)** Ryanodine (50 μM, 19 cells/3 dishes), and **(B)** Xestospongin C (10 μM, 14 cells/3 dishes). Ryanodine but not Xestospongin C inhibits Epac-agonist induced cytosolic calcium mobilization and calcium uptake in mitochondria. Arrow (↑) indicates application of agonist in each time course. F/F_0_ is the fractional change in fluorescence emission of the fluorescent probe or biosensor. Dash lines are standard error.

Moreover, the Me-cAMP/AM induced cytosolic and mitochondrial Ca^2+^ responses were completely blocked by ryanodine but were unaffected by xestospongin C (Figures 4A, B), indicating that the Epac-agonist mediated Ca^2+^ release was mainly derived from RyR-gated Ca^2+^ store to trigger mitochondrial Ca^2+^ transfer.

### ATP-mediated Ca^2+^ mobilization and Ca^2+^ transfer between ER and mitochondria

**FIGURE 5:**
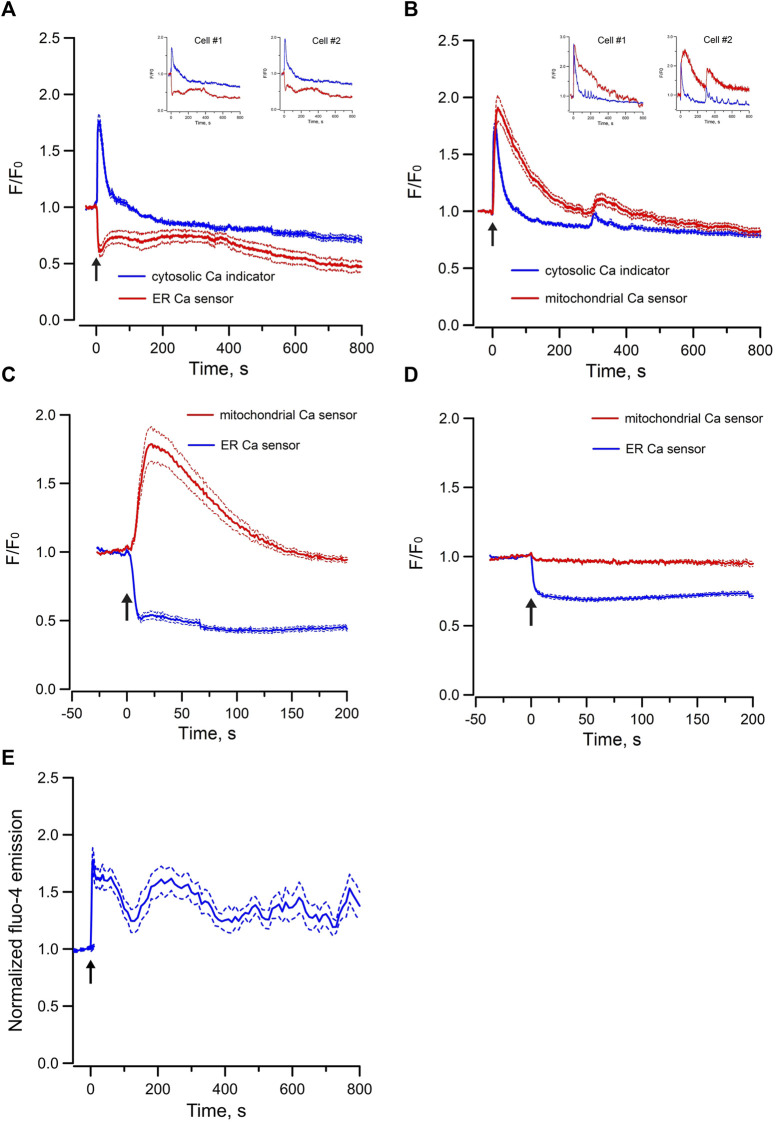
Mean normalized time courses of simultaneous changes in **(A)** cytosolic Ca^2+^ and ER luminal Ca^2+^ (15 cells/3 dishes), **(B)** cytosolic Ca^2+^ and mitochondrial matrix Ca^2+^ (9 cells/2 dishes), **(C)** ER luminal Ca^2+^ and mitochondrial matrix Ca^2+^ (32 cells/4 dishes) induced by 5 µM ATP, and **(D)** ER luminal Ca^2+^ and mitochondrial matrix Ca^2+^ induced by 200 µM Bt_3_-Ins(1,3,5)P_3_/AM in mpkCCD cells (45 cells/7 dishes). **(E)** mean normalized time course of cytosolic Ca^2+^ induced by 5 µM ATP in freshly isolated perfused rat IMCD (23 cells/3 tubules). The Arrow (↑) indicates application of agonist in each time course. Inserts are tracings from individual cells with multiple Ca^2+^ spikes. F/F_0_ is the fractional change in fluorescence emission of the fluorescent probe or biosensor. Dash lines are standard error.

In contrast, exogenous application of Bt_3_-Ins(1,3,5)P_3_/AM only triggered decrease in ER luminal Ca^2+^ without concomitant increase of mitochondria Ca^2+^ in mpkCCD cells (Figure 5D). To test whether increasing the abundance of endogenous IP_3_ can induce Ca^2+^ transfer between ER and mitochondria, ATP was used to stimulate endogenous IP_3_ production via purinergic receptors expressed in mpkCCD cells (Wildman et al., 2009). Application of ATP elicited a transient increase in cytosolic [Ca^2+^] of less than 50 s in mpkCCD cells (Figure 5A). Of note, this was associated with a sustained decrease ER luminal Ca^2+^ (Figure 5A), and a more prolonged increase in mitochondrial [Ca^2+^] (Figure 5B). When ER luminal Ca^2+^ and mitochondrial matrix Ca^2+^ were monitored simultaneously, ATP triggered decrease in ER luminal Ca^2+^ and concomitant increase in mitochondrial matrix Ca^2+^ (Figure 5C). These observations suggest that endogenous activation of IP_3_Rs is capable of triggering ER-mitochondrial Ca^2+^ transfer in mpkCCD cells, even though Epac-induced Ca^2+^ response is independent of the IP_3_R-dependent mechanism. ATP-induced intracellular Ca^2+^ mobilization was also detected in perfused IMCD (Figure 5E), which is consistent with the observations on ATP mobilized intracellular Ca^2+^ in mpkCCD cells.

### Epac-mediated store-operated Ca^2+^ entry in mpkCCD cells

**FIGURE 6:**
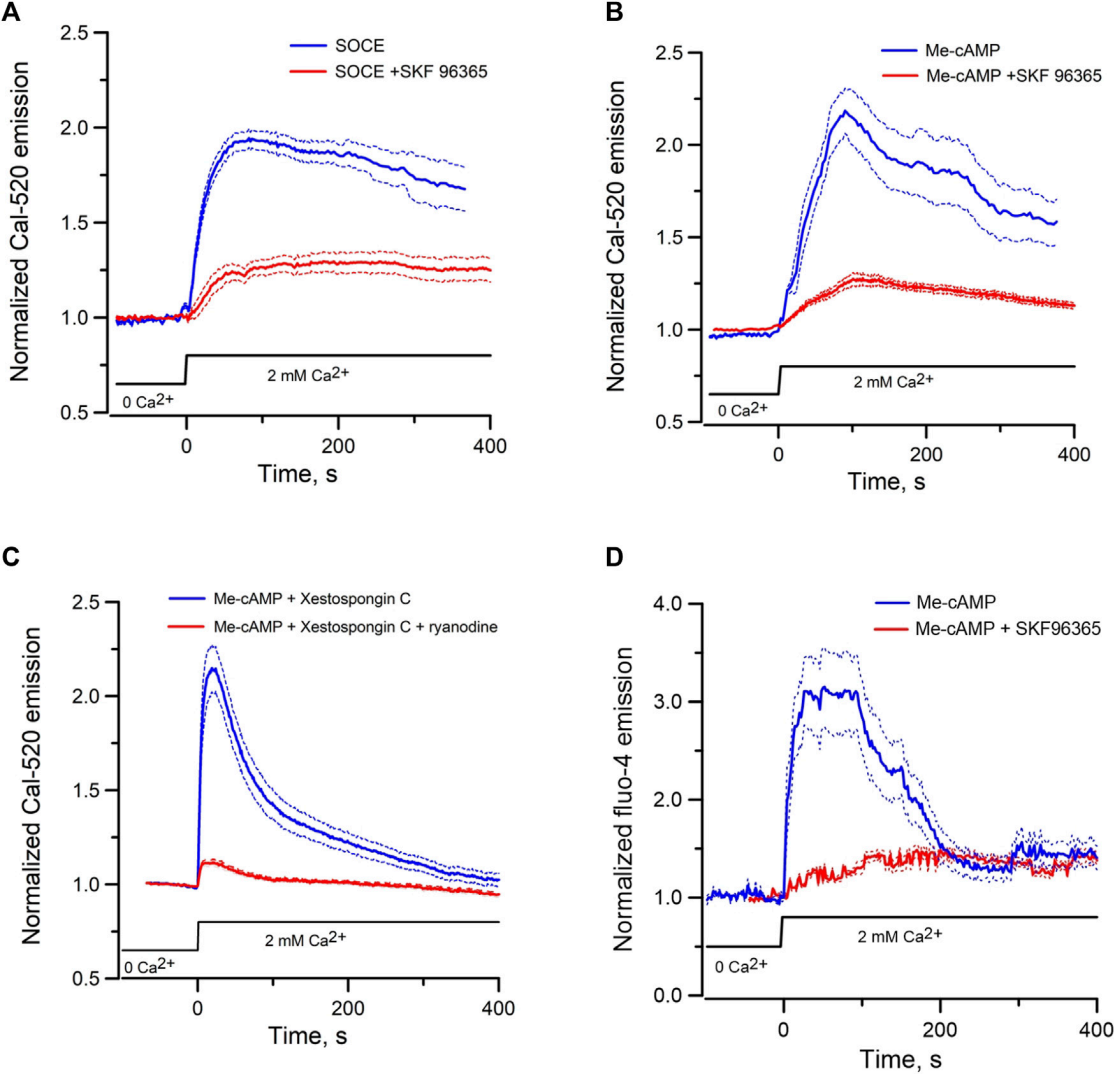
Mean normalized time courses of Ca^2+^ entry in mpkCCD cells and perfused IMCD triggered by re-addition of 2 mM of Ca^2+^. **(A)** Store-operated Ca^2+^ entry in the absence (69 cells/4 dishes) or presence (72 cells/3 dishes) of 50 µM SKF 96365 in mpkCCD cells, **(B)** Ca^2+^ entry induced by pre-incubation of mpkCCD cells with Epac-agonist (40 µM Me-cAMP/AM) in the absence (52 cells/3 dishes) or presence (59 cells/3 dishes) of SKF 96365, **(C)** Ca^2+^ entry induced by pre-incubation of mpkCCD cells with 40 µM Me-cAMP/AM in presence of 10 µM Xestospongin C (63 cells/3 dishes), or 10 µM Xestospongin +50 µM ryanodine (79 cell/3 dishes). **(D)** Ca^2+^ entry induced by pre-incubation of perfused IMCD with 40 µM Me-cAMP/AM in the absence (39 cells/4 tubules) or presence (27 cells/3 tubules) of SKF96369. Dash lines are standard error.

Since Epac-activation mobilizes ER Ca^2+^ to elicit cytosolic Ca^2+^ signals and ER-mitochondrial Ca^2+^ transfer, the reduction in ER luminal Ca^2+^ may activate SOCE to replenish ER Ca^2+^ stores. SOCE was first examined in mpkCCD by depleting ER Ca^2+^ stores using the SERCA inhibitor thapsigargin (10 µM) in the absence of extracellular Ca^2+^. Reintroduction of 2 mM Ca^2+^ in the external solution elicited robust Ca^2+^ entry, which was inhibited by the SOCE inhibitor SKF96365 (50 µM) (Figure 6A). Incubation of mpkCCD cells with the Epac-agonist Me-cAMP/AM in the absence of extracellular Ca^2+^ also activated SOCE similar to that induced by thapsigargin (Figure 6B). Robust Me-cAMP/AM induced Ca^2+^ entry was also observed in mpkCCD cells pretreated with xestospongin C; but the response was abolished in cells treated with both ryanodine and xestospongin C (Figure 6C). Moreover, Me-cAMP/AM-induced Ca^2+^ entry was observed in isolated perfused IMCD, and the effect was abolished by inhibition of SOCE using SKF96365 (Figure 6D). These results suggest that Epac activation is capable of inducing ER luminal Ca^2+^ depletion and SOCE in both mpkCCD and perfused IMCD.

## Discussion

Epac-dependent Ca^2+^ mobilization was associated with AVP-induced apical exocytosis in perfused IMCD. mpkCCD cells have been used as the cell model for transcriptomic and phosphoproteomic studies of AVP-signaling in the collecting duct (Rinschen et al., 2010; Huling et al., 2012; Sandoval et al., 2013; Yang et al., 2022; Park et al., 2023). We have demonstrated that mpkCCD cells retain the characteristics of Epac-dependent Ca^2+^ mobilization as in intact IMCD cells. Taking advantage of expressing ER and mitochondrial specific biosensor proteins in mpkCCD cells, the dynamic properties and relationship between cytosolic Ca^2+^, ER luminal Ca^2+^, and mitochondrial matrix Ca^2+^ were characterized. Epac-agonist mobilized Ca^2+^ from ER Ca^2+^ stores, depleted ER luminal Ca^2+^, and activated SOCE in mpkCCD cells. The oscillation of cytosolic Ca^2+^ triggered by Epac-agonist was entrained to Ca^2+^ oscillation in mitochondrial matrix, while 180° out-of-phase to the oscillation in ER luminal Ca^2+^. These observations indicated that Epac-mediated oscillatory Ca^2+^ signaling event is an integrated process which involves interplay of luminal ER Ca^2+^ release and refill, Ca^2+^ entry and efflux in mitochondrial matrix, and extracellular Ca^2+^ entry secondary to ER Ca^2+^ depletion in mpkCCD cells.

Time series of Ca^2+^ oscillation extracted from mpkCCD cells were analyzed in frequency domain using algorithm based on Fast Fourier Transform. Frequencies of Epac-dependent oscillations were detected in the range between 0.007 and 0.1 Hz, which is similar to the frequency ranges induced by cADP-ribose, an endogenous agonist of RyRs, observed in intact IMCD cells (Yip, 2002; Yip and Sham, 2011). Moreover, the sustained Me-cAMP-mediated Ca^2+^ oscillation in mpkCCD cells is similar to those observed in perfused IMCD triggered by caged cyclic-ADP-ribose (Yip and Sham, 2011). However, the 4-CMC-induced Ca^2+^ transient is more transient in the IMCD cells. The disparity in the kinetic profile of 4-CMC-triggered Ca^2+^ oscillations could be related to the differences in the agonist sensitivity, the activation/inactivation kinetics, sensitization of Ca^2+^-induced-Ca^2+^ release of the RyRs. Nevertheless, the complete inhibition of Epac-agonist-induced Ca^2+^ oscillation with ryanodine, but not by the IP_3_R-antagonist xestrospongin C, suggests that RyR is the primary Ca^2+^ source contributing to the Epac-induced Ca^2+^ oscillation in the mpkCCD cells.

It has been established that AVP regulates AQP2 shuttling through a cAMP-dependent pathway, and PKA has been considered as the only effector protein of cAMP for mediating AVP-regulated water permeability in kidney collecting ducts (Knepper and Inoue, 1997). It is now known that Epac is an important effector protein of cAMP. Epac1 and Epac2 isoforms are expressed in collecting duct and mpkCCD cells (Li et al., 2008; Kortenoeven et al., 2012). Our previous study found that Epac activation, but not PKA activation, mimics AVP in triggering Ca^2+^ mobilization and oscillations and induces apical shuttling of AQP2 in perfused IMCD (Yip, 2006). In the present study, PKA specific cAMP analog (6-Bnz-cAMP/AM) did not trigger Ca^2+^ oscillations in mpkCCD cells, while Epac-specific cAMP (Me-cAMP/AM) triggered long lasting Ca^2+^ oscillations. Moreover, long-term regulation of AQP2 by AVP in mpkCCD cells is mediated by Epac but not by PKA (Kortenoeven et al., 2012). Such evidence is consistent with an Epac-dependent signal pathway for regulation of collecting duct water permeability. It is also consistent that mice lacking Epac1 or Epac2 showed impaired urinary concentration ability and augmented urinary excretion of Na^+^ and urea (Cherezova et al., 2019). However, no defects in AVP-induced Ca^2+^ signaling in split-opened collecting ducts or changes in AQP2 protein abundance were observed. The urinary concentrating defect might be caused by reduced expression of the Na^+^/H^+^ exchanger isoform 3 (NHE3) (Cherezova et al., 2019). An inhibitory effect of Epac1 on NHE3 activity was previously shown in opossum kidney cells and mouse kidney slices (Honegger et al., 2006). A recent study reported compromised tight junctions in the collecting duct of Epac1 knockout mice in conjunction with a reduced papillary osmolarity (Sivertsen Asrud et al., 2020), suggesting Epac1 is involved in regulating paracellular permeability in the collecting duct. The reason for the discrepancies in the two knock-out mice studies is unclear but could be related to mouse dietary conditions or the existence of a different microbiome between different institutions.

Our data also show that Epac-agonist triggered Ca^2+^ oscillations resemble those activated by a RyR agonist in mpkCCD cells, suggesting that Epac-agonist mobilizes ER Ca^2+^ in mpkCCD cells via RyRs. Organelle-specific Ca^2+^-sensitive biosensors expressed in mpkCCD cells showed that the Epac agonist triggered synchronized Ca^2+^ spikes in cytosol and mitochondrial matrix, which are temporally correlated with reciprocal changes in ER luminal Ca^2+^. These observations indicated that agonist-induced Epac activation not only triggered release of Ca^2+^ into cytosol, but also transferred Ca^2+^ from ER to mitochondria.

The resting [Ca^2+^] of mitochondrial matrix is comparable to the resting cytosolic [Ca^2+^]. Mitochondrial Ca^2+^ uptake takes place at specialized microdomains where cytosolic [Ca^2+^] are high. Such microdomains are localized in the mitochondria-associated membranes (MAMs), where the endoplasmic reticulum membrane is within 10–30 nm from the outer mitochondrial membrane (Hajnoczky et al., 2002). Ca^2+^ released from ER enters the intermembrane space through voltage-dependent anion channels (VDACs) localized in the outer membrane, and then enters mitochondrial matrix through the mitochondrial Ca^2+^ uniporter (MCU) of the inner membrane (Patergnani et al., 2011; Giorgi et al., 2015). RyR agonist (4-CMC) triggered similar Ca^2+^ transfer from ER to mitochondria as Epac-agonist suggested that the Epac-induced ER-mitochondrial Ca^2+^ transfer is mediated by RyRs in MAMs of mpkCCD cells. IP_3_Rs and RyRs have been localized in MAMs (Hajnoczky et al., 2002; Chen et al., 2012; Bartok et al., 2019), but their distribution in mpkCCD cells or native renal collecting duct cells is unclear. The current study explored on this knowledge gap. Exogenous Bt_3_-Ins(1,3,5)P_3_/AM, which effectively activated cytosolic Ca^2+^ oscillation, only triggered a decrease in ER luminal Ca^2+^ but not concomitantly increased mitochondria Ca^2+^ in mpkCCD cells. It is possible that IP_3_Rs are less efficacious in facilitating ER-mitochondrial Ca^2+^ transfer in mpkCCD cells; or the cell permeant Bt_3_-Ins(1,3,5)P_3_/AM, had a poor access to the IP_3_Rs in MAMs. To test the latter hypothesis, the native agonist ATP was applied to increase endogenous IP_3_ abundance in mpkCCD cells. ATP triggered synchronized increase in cytosolic [Ca^2+^] with concomitant elevation in mitochondrial matrix [Ca^2+^] and depletion ER luminal Ca^2+^. ATP-induced Ca^2+^ transfer between ER and mitochondria was also visualized in cells co-expressed with ER and mitochondrial Ca^2+^ biosensors. These observations indicated that IP_3_Rs is capable of mediating Ca^2+^ transfer between ER and mitochondria in mpkCCD cells. However, the kinetics of ATP-induced cytosolic, endoplasmic, and mitochondrial Ca^2+^ responses are distinctly different from those induced by Epac or RyR agonists, distinguishing the Ca^2+^ signals activated by the two different agonists induced signaling pathways. Our studies also support that this mechanism is at play *in vivo* and it is noteworthy that 5 µM ATP also triggered Ca^2+^ mobilization and oscillations in isolated perfused IMCD (Figure 5E), confirming that purinergic receptor mediated Ca^2+^ mobilization is present in both intact IMCD cells and mpkCCD cells. Consistent with this, studies in acutely isolated connecting tubule/collecting duct of mice support that an acute increase in cytosolic [Ca^2+^] inhibits ENaC activity (Mamenko et al., 2011) mediated by P2Y_2_ receptor activation (Pochynyuk et al., 2008).

Mitochondrial Ca^2+^dynamics plays important roles in intracellular Ca^2+^signaling, cell metabolism, cell survival, and other cell-type specific functions (Rizzuto et al., 2012). As described above MCU supports cytoplasmic Ca^2+^ oscillations, SOCE and Ca^2+^-dependent gene expression in response to receptor-mediated stimulation (Samanta et al., 2014). It has been proposed that mitochondrial Ca^2+^ shuttling via MCU sustains the cytosolic Ca^2+^ signal by preventing Ca^2+^-dependent inactivation of IP_3_Rs and store-operated CRAC channels (Yoast et al., 2021). ER-mitochondrial Ca^2+^ transfer also stimulates Ca^2+^-sensitive dehydrogenases and respiratory chain components to promote oxidative phosphorylation, ATP, and ROS production (Territo et al., 2000; Territo et al., 2001; Hou et al., 2013). The physiological implications for the ER-mitochondrial Ca^2+^ transfer in the regulation of cellular functions in renal tubular cells remain to be determined.

In conclusion, mpkCCD cells retained all reported features of Epac-induced Ca^2+^ mobilization observed in isolated perfused IMCD. The temporal relationship between cytosolic Ca^2+^, ER luminal Ca^2+^, and mitochondrial matrix Ca^2+^ activated by Epac and RyR-agonists are highly compatible, but is distinctly different from those induced by IP_3_R stimulation. Furthermore, we have provided the first characterization of ER-mitochondrial Ca^2+^ transfer in mpkCCD cell, which can be used as a renal cell model to address novel questions of how mitochondrial Ca^2+^ regulates cytosolic Ca^2+^ signals, inter-organellar Ca^2+^ signaling, and other renal tubular functions.

## Data Availability

The raw data supporting the conclusions of this article will be made available by the authors, without undue reservation.
